# Rhythmic contraction but arrhythmic distension of esophageal peristaltic reflex in patients with dysphagia

**DOI:** 10.1371/journal.pone.0262948

**Published:** 2022-01-24

**Authors:** Kazumasa Muta, Ravinder K. Mittal, Ali Zifan

**Affiliations:** Division of Gastroenterology, Department of Medicine, University of California San Diego, San Diego, CA, United States of America; Sapienza University of Rome, ITALY

## Abstract

**Background:**

Reason for dysphagia in a significant number of patients remains unclear even after a thorough workup. Each swallow induces esophageal distension followed by contraction of the esophagus, both of which move sequentially along the esophagus. Manometry technique and current system of classifying esophageal motility disorders (Chicago Classification) is based on the analysis of the contraction phase of peristalsis.

**Goal:**

Whether patients with unexplained dysphagia have abnormalities in the distension phase of esophageal peristalsis is not known.

**Methods:**

Using Multiple Intraluminal esophageal impedance recordings, which allow determination of the luminal cross-sectional area during peristalsis, we studied patients with nutcracker esophagus (NC), esophagogastric junction outflow obstruction (EGJOO), and functional dysphagia (FD).

**Results:**

Distension contraction plots revealed that swallowed bolus travels significantly faster through the esophagus in all patient groups as compared to normals. The luminal cross-sectional area (amplitude of distension), and the area under the curve of distension were significantly smaller in patients with NC, EGJOO, and FD as compared to normals. Bolus traverses the esophagus in the shape of an “American Football” in normal subjects. On the other hand, in patients the bolus flow was fragmented. ROC curves revealed that bolus flow abnormalities during peristalsis are a sensitive and specific marker of dysphagia.

**Conclusion:**

Our findings reveal abnormality in the distension phase of peristalsis (a narrow lumen esophagus) in patients with dysphagia. We propose that the esophageal contraction forcing the swallowed bolus through a narrow lumen esophagus is the cause of dysphagia sensation in patients with normal contraction phase of peristalsis.

## Introduction

Dysphagia or difficulty swallowing is common in the general population (~ 15%) [1]. It is generally classified as oropharyngeal and esophageal, both of which can be due to the pathologies that range from intrinsic or extrinsic to the respective anatomical structures. In the case of esophageal dysphagia, intrinsic pathologies such as cancer, stricture, rings, webs and eosinophilic esophagitis can be identified easily by upper endoscopy/biopsy and or barium swallow studies. Esophageal manometry (high resolution manometry) identifies disorders of esophageal peristalsis, such as achalasia esophagus, nutcracker/jackhammer, distal esophageal spasm, and others in patients with dysphagia [2]. Using high resolution manometry (HRM) and strict criteria to distinguish normal versus abnormal (Chicago Classification) contraction parameters, 34% of patients with dysphagia were found to have normal manometry study [3]. Rome classification defines these patients as having “functional dysphagia” (FD) [4], which implies hypersensitivity or dysfunctional sensory/cognitive system in these patients.

Each swallow induces esophageal distension followed by contraction, both of which travel from the top to the bottom of esophagus in a sequential/peristaltic fashion [5, 6]. The current system of classifying esophageal motility disorders (Chicago classification) is based the parameters of LES relaxation and contraction phase of peristalsis [7]. Distension of the esophagus by the swallowed bolus is a surrogate marker of the inhibition phase of peristalsis. Multiple Intraluminal esophageal impedance recordings allow measurement of the luminal cross-sectional area (CSA) during peristalsis, i.e., esophageal distension [8, 9]. The goal of our study was to compare the distension-contraction profile of esophageal peristalsis in patients with dysphagia, who meet criteria for NC esophagus, EGJOO and FD. We used a custom developed computer software program to determine the distension amplitude and temporal relationship between distension and contraction during swallow-induced peristalsis.

## Methods

### Study population

We studied 4 groups of subjects, 24 healthy asymptomatic normal subjects (age, 34 ±12 years, 10 males), 24 patients with nutcracker esophagus (NC), (age 62 ± 9 years, 9 males), 24 patients with Functional Dysphagia (FD) (age 54 ± 13, 6 males) and 24 patients with symptomatic EGJOO (age 69 ± 13, 7 Males). All patients were referred to the University of California San Diego, GI function laboratory for assessment of esophageal dysphagia. The study was approved by the Human Research Protection Program of the University of California, San Diego and all subjects signed an informed consent prior to participation in the study (protocol # 091745). Written informed consent was obtained from the patient of the study, witnessed by the nurses in charge of data acquisition. None of the normal subjects were taking any medications known to affect the esophagus or swallowing function. The criteria for defining various groups were based on the HRMZ recordings performed using the Manoscan system (Medtronics Inc., Minneapolis, MN) and interpreted using Chicago Classification (3.0) of esophageal motility disorders. The data from some normal subjects [6, 10], NC patients and FD [11] patients have been reported earlier [12]. The NC esophagus was defined, when the mean contraction amplitude of 10 swallows at any location in the distal esophagus was > 200mmHg. FD was the diagnosis of exclusion (normal upper endoscopy, esophageal biopsy and HRM study). EGJOO was the diagnosis in patients with impaired relaxation of the esophagogastric junction (lower esophageal sphincter) with an integrated relaxation pressure (IRP) of > 15mmHg, and normal parameters of esophageal contraction and peristalsis. In addition to the above, all patients had a normal endoscopy and biopsy. The human investigation committee of the University of California, San Diego approved the study protocol (IRB # 182156 & 202106X). Subjects completed standardized questionnaire documenting age, sex, body mass index (BMI), and symptoms prior to the manometry laboratory. Dysphagia score was assessed using a standardize and validate, brief esophageal dysphagia scoring (BEDS) questionnaire [13]. All patients were selected from a large cohort of patients studied who met criteria, as specified above, for each group.

### HRMZ recordings

Subjects were studied using a catheter assembly that consisted of an HRMZ catheter (4.2mm diameter; Medtronic Inc., MN), equipped with 36 pressure transducers (1 cm apart), and 18 impedance electrodes (2 cm apart). Viscous lidocaine (2% lidocaine hydrochloride topical solution, USP) was administered orally and nasally for local anesthesia, followed by placement of the catheter through the nose. A routine clinical manometry study protocol (patients in the supine position and 10 swallows, each of 5ml, 0.5N saline at room temperature) was followed by tilting the stretcher to -15^o^ degree (Trendelenburg position) and 8–10 swallows of 10 ml, 0.5N saline warmed to 37^0^ Celsius (see S1 Fig). The later protocol allows accurate measurement of the luminal CSA from the impedance part of HRMZ recording [9].

### Data analysis

The HRMZ recording were analyzed using Dplots [14] (Motilityviz, La Jolla, CA.), a MATLAB based program (Mathworks, Natick, Massachusetts, USA). After importing HRMZ studies into the Dplots, individual swallows (7–10 in each subject) were selected for analysis. A rectangular region of interest (ROI), encompassing the time from the onset of upper esophageal sphincter relaxation to 2 seconds after the return of LES pressure to baseline (at the end of peristaltic contraction) was selected. The selected ROI was located between the lower border of the UES and upper edge of LES. The selected ROI was divided into 4 equal segments, and following parameters were extracted automatically by the Dplots software for each segment, (1) peak amplitude of distension (CSA), (2) area under the curve (AUC) of distension, (3) peak amplitude and AUC of contraction, (3) time between the onset of UES relaxation and peak distension (T1), and (4) time between the onset of UES relaxation and peak contraction (T2).

Statistical hypothesis testing was used to study the differences among the 4 groups. The goal was to study the importance and relevance of pressure and impedance derived features in discriminating between groups. Due to the multi-class nature of the problem (i.e., 5 classes), we used a distributed output approach, namely the Error-Correcting Output Codes (ECOC) method [15, 16], which is an ensemble method that allows a multi-class problem to be reframed as a multiple binary classification problem. Combined with boosting (a general method of improving the accuracy of a given base or “weak” learning algorithm), we employed an ensemble of (decision) trees [17] as the binary learners. The training set for each base classifier is based on bootstrapping sampling, i.e., random selection of data set with replacement (replacement means same data can be chosen many times in the same subset). During testing of a new variable, the classification is completely based on the simple majority voting scheme, i.e., each classifier predicts a class for the instance, and the class that has the maximum number of votes cast by each base classifier is declared as the final class. We used the approach of one-vs-all (OVA) technique, which allows estimation of the better posterior probabilities [18]. The output representations improve the generalization performance of multiclass learning tasks for unseen data. Finally, 10-fold cross validation (a resampling procedure used to evaluate machine learning models on a limited data sample), was applied to study the generalization power (or skill) of the model for unseen data.

### Statistical analysis

A power analysis [19] was conducted a priori to estimate the sample size for this study. For the omnibus null hypothesis, based on a Cohen’s *f* of 0.445 (distal peak distension difference of Normal and FD patients [20], assuming similar distribution among the nutcracker and the EGJOO patient groups), indicated that for the omnibus null hypothesis with an α  =  0.05, we would need 60 subjects split equally across the 4 groups to power the effect at 80%. The sample size was further increased to power the post-hoc pairwise comparisons order to reach statistical power of 0.80 for the non-null comparisons for a total sample size of 96 (24 in each group). Quantitative data are reported as mean ± standard deviation (unless otherwise stated). A Shapiro-Wilk’s test was used to assess normality of distribution before running parametric tests. For continuously measured variables, 1-way analyses of variance (ANOVA) were conducted. If Bartlett test of homogeneity of variances was non-significant (P ≥ 0.05) for a given variable, a Tukey post hoc analysis was conducted. If Bartlett test was significant (P < 0.05), Welch t-tests (accommodating unequal variances) were conducted, with Holm-Bonferroni adjustment for multiple comparisons. All tests were 2-tailed with α set at 0.05. Receiver operator characteristics (ROC) curves (one-vs-all classifier) were generated to determine sensitivity and specificity of the parameters in distinguishing normal subjects from the patient groups.

## Results

**Fig 1:**
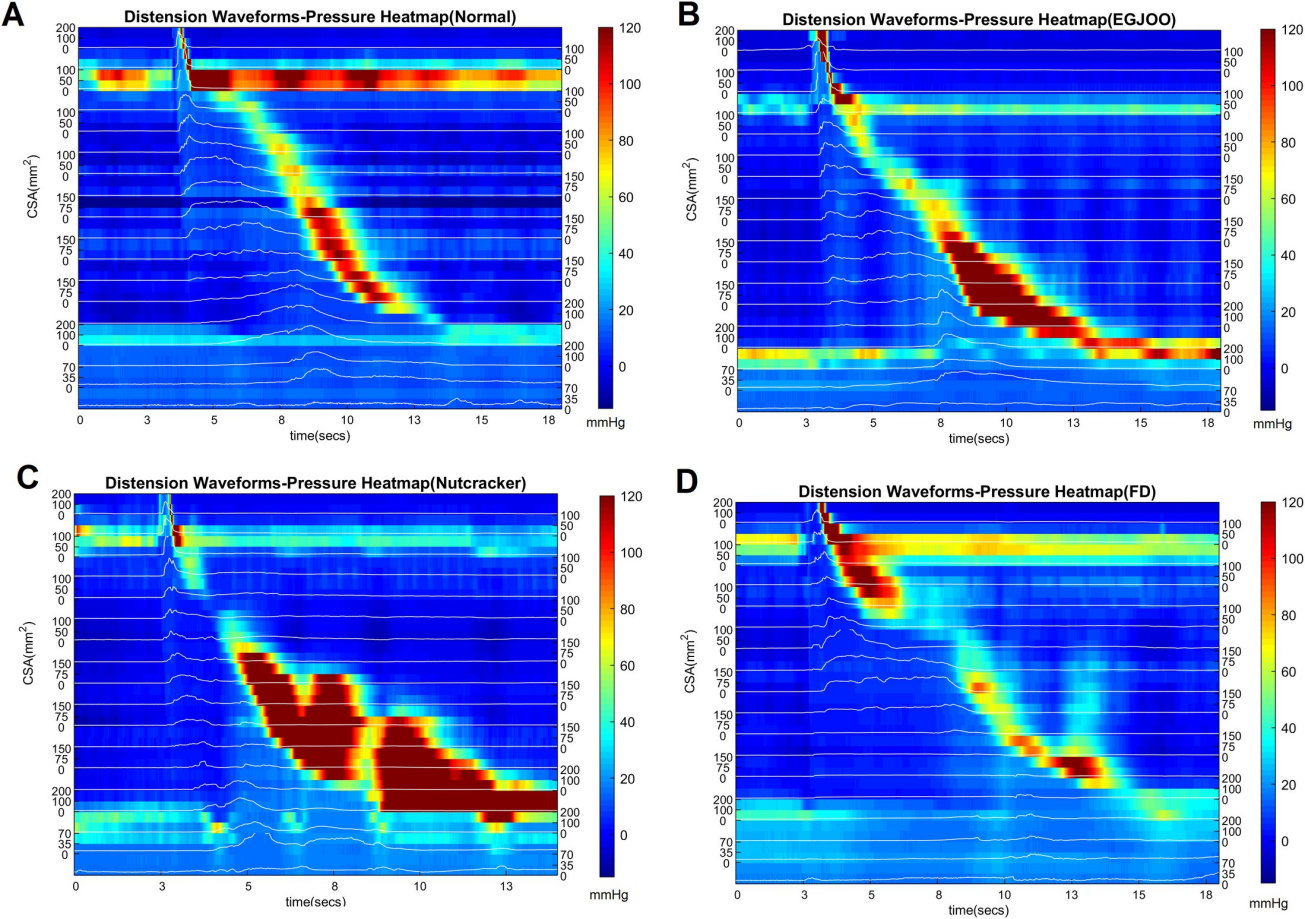
Pressure heatmaps of a (A) normal subject, (B) patient with esophagogastric junction outflow obstruction (EGJOO), (C) patient with nutcracker esophagus (NC), and (D) patient with functional dysphagia. Note, that the amplitude of distension in patients is smaller and there is alteration in the temporal relationship between distension and contraction.

**Fig 2:**
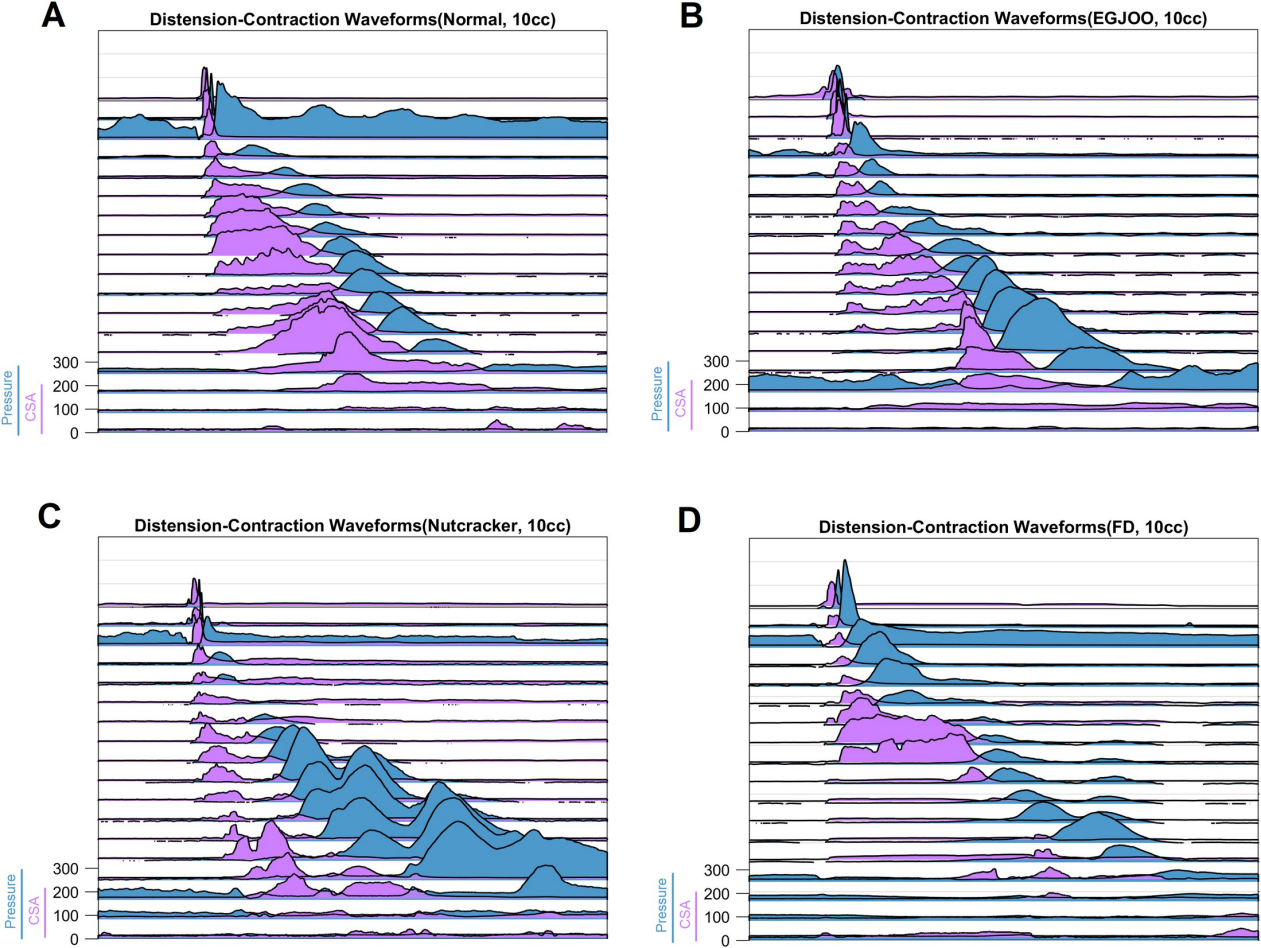
Distension-contraction plots of the pressure and impedance measurements of Fig 1. Both distension and contraction are displayed as waveforms in this figure. (A) normal subject, (B) patient with esophagogastric junction outflow obstruction (EGJOO), (C) patient with nutcracker esophagus (NC) and (D) patient with functional dysphagia. Note, that the amplitude of distension in patients is smaller and there is alteration in the temporal relationship between distension and contraction.

**Fig 3:**
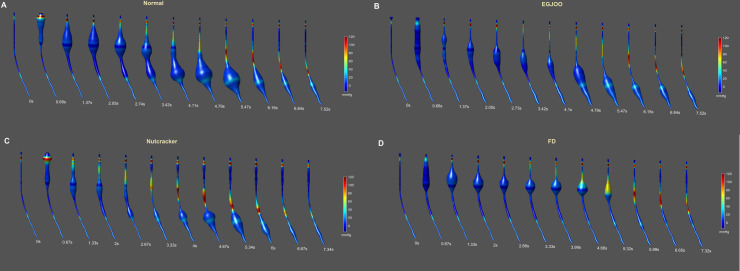
Schematic of distension contraction patterns along the length of the esophagus in, (A) normal subject, (B) patient with esophagogastric junction outflow obstruction (EGJOO), (C) patient with nutcracker esophagus (NC), and (D) patient with functional dysphagia. (same swallows from Figs 1 and 2). Note, that the amplitude of distension in patients is smaller and there is alteration in the temporal relationship between distension and contraction waveform.

**Fig 4:**
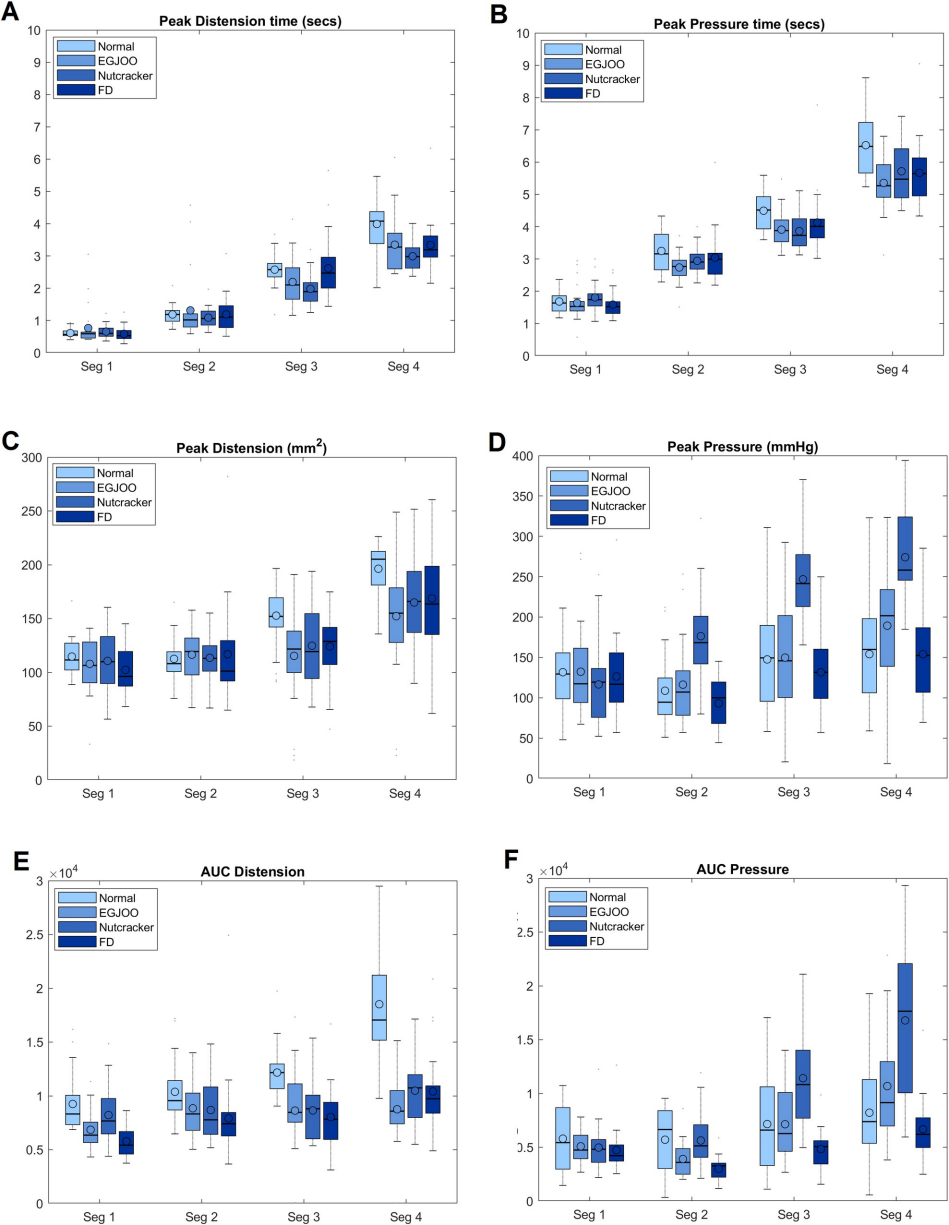
Boxplots of the distension contraction parameters in controls (normal subjects) and 3 patient groups: EGJOO = esophagogastric junction outflow obstruction, NC = nutcracker esophagus, FD = functional dysphagia. (A) time difference between the onset of swallow and distension amplitude, (B) time between the onset of swallow and peak contraction amplitude, (C) peak distension amplitude, (D) peak contraction amplitude, (E) Area under the curve (AUC) of distension and (F) AUC of pressure. Means are displayed as circles.

Figs 1 and 2 show the distension contraction pattern along the entire length of the esophagus, over time during a single swallow from a normal subject, patient with nutcracker esophagus (NC), a patient with functional dysphagia (FD) and a patient with esophagogastric junction outflow obstruction (EGJOO). In Fig 1, distension is shown as waveform and contraction as color topograph. In Fig 2, both distension and contraction are shown as waveforms to visualize the amplitudes and temporal relationship between distension and contraction. In normal subjects, distension precedes contraction at each location. The peak of distension and onset as well as peak of contraction traverses sequentially (peristaltic fashion) along the length of the esophagus. The end of distension wave is temporally related to the onset of contraction (complete bolus clearance with peristaltic contraction). To capture the temporal evolution of bolus movement through the esophagus, schematic of the bolus propulsion from a swallow, in a normal subject and 3 dysphagia patients (one from each group) is shown in Fig 3 (see also S1 Video). The distension waveform in normal subjects is a bell-shaped curve with a single peak. The distension amplitude and duration are greater in the distal as compared to proximal esophagus in normal subject. In patients, the pattern of distension differs from normal subjects in that, 1) the amplitude of distension is smaller, 2) bolus arrives in the distal esophagus faster in patients, and 3) distension waveform is multi-peaked. The latter implies that bolus moves in a fragmented fashion along the length of esophagus. Fig 4 shows average data of peak pressure time, peak distension time, peak amplitude of pressure and distension, and AUC of distension and pressure waveforms.

### Peak pressure time

The peak pressure time, which is reflective of latency of contraction after the onset of swallow was slightly but significantly lower in the 3 patient groups as compared to normal, especially in the most distal (i.e., 4^th^ segment). In the 3^rd^ segment, normal group (4.50±0.65 sec) was longer than the EGJOO (3.91± 0.56secs, 95% CI = 0.126 to 1.054; P = 0.0067) and Nutcracker patient (3.86 ± 0.57, 95% CI = 0.157 to 1.115; P = 0.0039). In the 4^th^ segment, the values were significantly lower in 3 patient groups as compared to normals. Normal: 6.52±0.99secs, EGJOO (5.36±0.84secs, 95% CI = 0.445 to 1.862; P = 0.0003), with Nutcracker (5.72±0.9secs, 95% CI = 0.0832 to 1.501; P = 0.0222) and FD: 5.67±1.03, 95% CI = 0.130 to 1.548; P = 0.0135).

### Peak distension time

The peak distension time was significantly shorter in 3 patient groups as compared to normals, more so in the 4^th^ segment (normal, 3.99±0.78secs), (EGJOO, 3.35±0.85 secs, 95% CI = 0.0224 to 1.236; P = 0.0391), NC, 2.99±0.43secs, 95% CI = 0.4867 to 1.4863; P<0.0001), and FD, 3.34±0.81secs, 95% CI = 0.027 to 1.248; P = 0.0367). There was significant difference in the 3^rd^ segment between normal subjects and nutcracker group. With regards to difference between 3 groups, nutcracker group had shorter durations compared to the FD groups.

### Peak pressure

As expected, peak pressure was higher in the nutcracker patients compared to normal subjects as well as with other patient groups in all segments. There were no differences between normal subjects and patients with EGJOO and FD.

### Peak distension

The distension amplitude was significantly lower in patients as compared to normal subjects in the 3^rd^ and 4^th^ segment of the esophagus. 3^rd^ segment values were, normal:153±31mm^2^, EGJOO:115±44mm^2^, 95% CI = 7.9 to 68.9; P = 0.0069), nutcracker: 125±36mm^2^, 95% CI, 3.023 to 54.839; P = 0.0217), and FD:124± 33 mm^2^, 95% CI = 0.632 to 55; P = 0.0429). The 4^th^ segment values for peak distension, normal: 196 ± 25 mm^2^, EGJOO: 152±53 mm^2^, 95% CI, 11.008 to 79.29; P = 0.0059, nutcracker: 165±36 mm^2^, 95%CI = 7.034 to 55.716; P = 0.0076), and FD: 169±45 mm^2^, 95% CI, 0.702 to 56.4362; P = 0.0496). No differences were observed between the 3 patient groups.

### AUC distension

The difference between normal subjects and patients were significantly greater and marked in the 3^rd^ and 4^th^ segments. The 3^rd^ segment values for AUC distension were, normal:12168 ±2433, EGJOO:8633 ± 4297, 95% CI = 779 to 6078; P = 0.00657, nutcracker: 8647 ± 2695, 95% CI = 1422 to 5408; P = 0.00017, and FD: 8038± 2773, 95% CI = 1946.303 to 6102.97; P<0.0001. The 4^th^ segment values for AUC of distension were normal:18518 ± 5068 mm^2^, EGJOO: 8754±3625 mm^2^, 95%CI, 6538 to 12451; P<0.0001, nutcracker: 10485±2917, 95%CI = 4881 to 10793; P<0.0001), and FD: 10411±3648, 95% CI = 4881 to 10793; P < .0496.

### Classification performance measure

**Fig 5:**
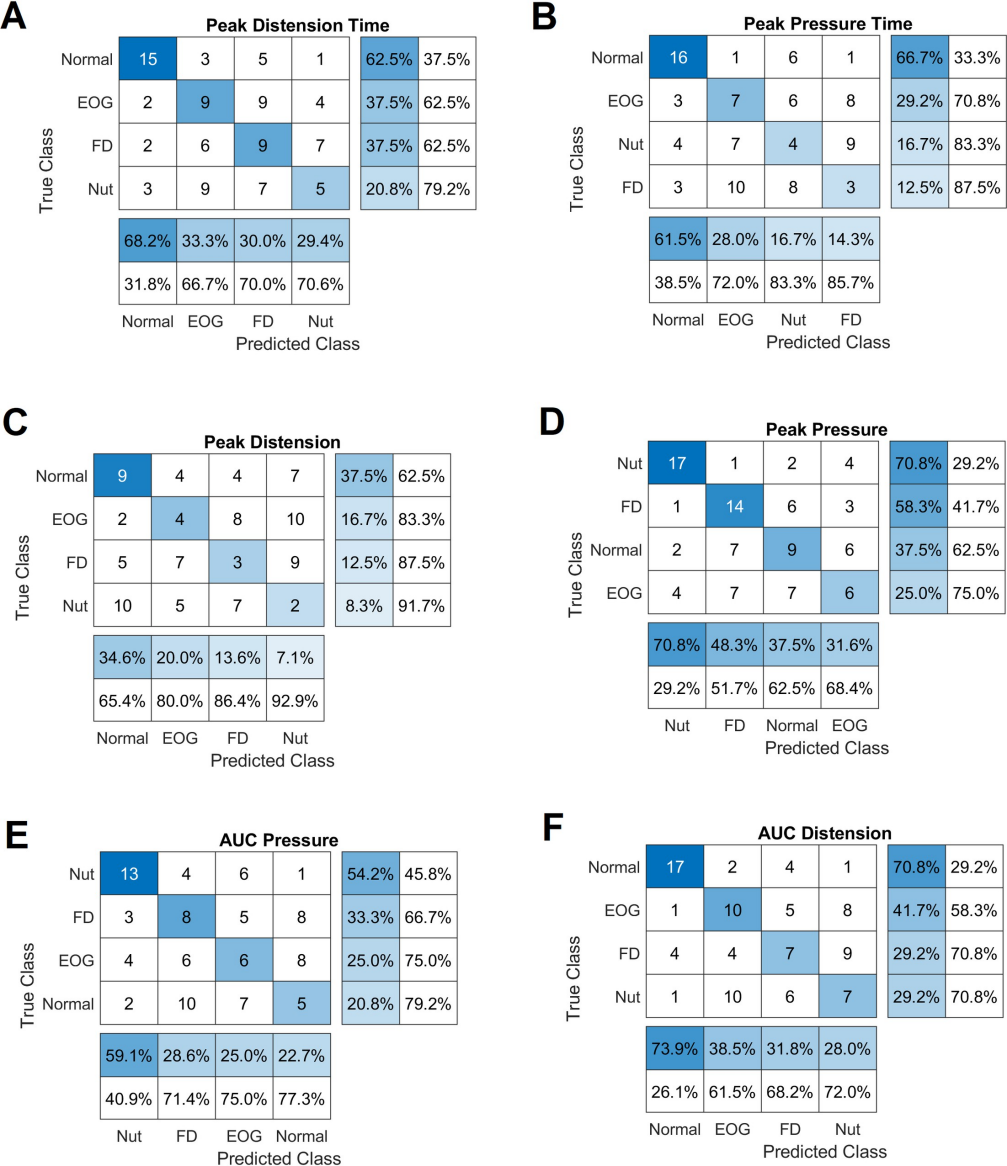
Classification results from 10-fold cross validation, using combination of single and paired (best performing) features. In the tables, EGJOO group has been denoted by EOG. Note, confusion matrix has been sorted by class-wise recall (true positive rate) in columns, and (row-wise) by class-wise precision (positive predictive value).

The degree of a classifier performance is measured by the confusion matrix, by comparing the predicted labels by the classifier and actual label of classes. The classification result for the (10-fold cross-validated) discrimination of the four groups are shown in the tables of Fig 5 for each of the features. The last two rows in the confusion matrix shows the True positive rate (TPR or sensitivity) of the classifier for each class and the last columns represent positive predicted values (PPV). The results indicate the superiority of the accuracy of AUCD (PPV = 70.8% & TPR = 73.9%) in separating the 4 groups. The same is also true for peak pressure in the Nutcracker group. Further statistical (i.e., McNemar) tests, were carried out to test whether the peak distension time and peak pressure time had equal predictive accuracies on the test sets. However, that was not the case for the two features (P = 0.175). Moreover, similar results were obtained comparing AUC distension with Peak distension time(P = 0.617), and peak pressure time (P = 0.088), underscoring the equal importance of 3 features in the normal group compared to the rest of patient groups.

### Subjective dysphagia symptom scores versus objective measures

**Fig 6:**
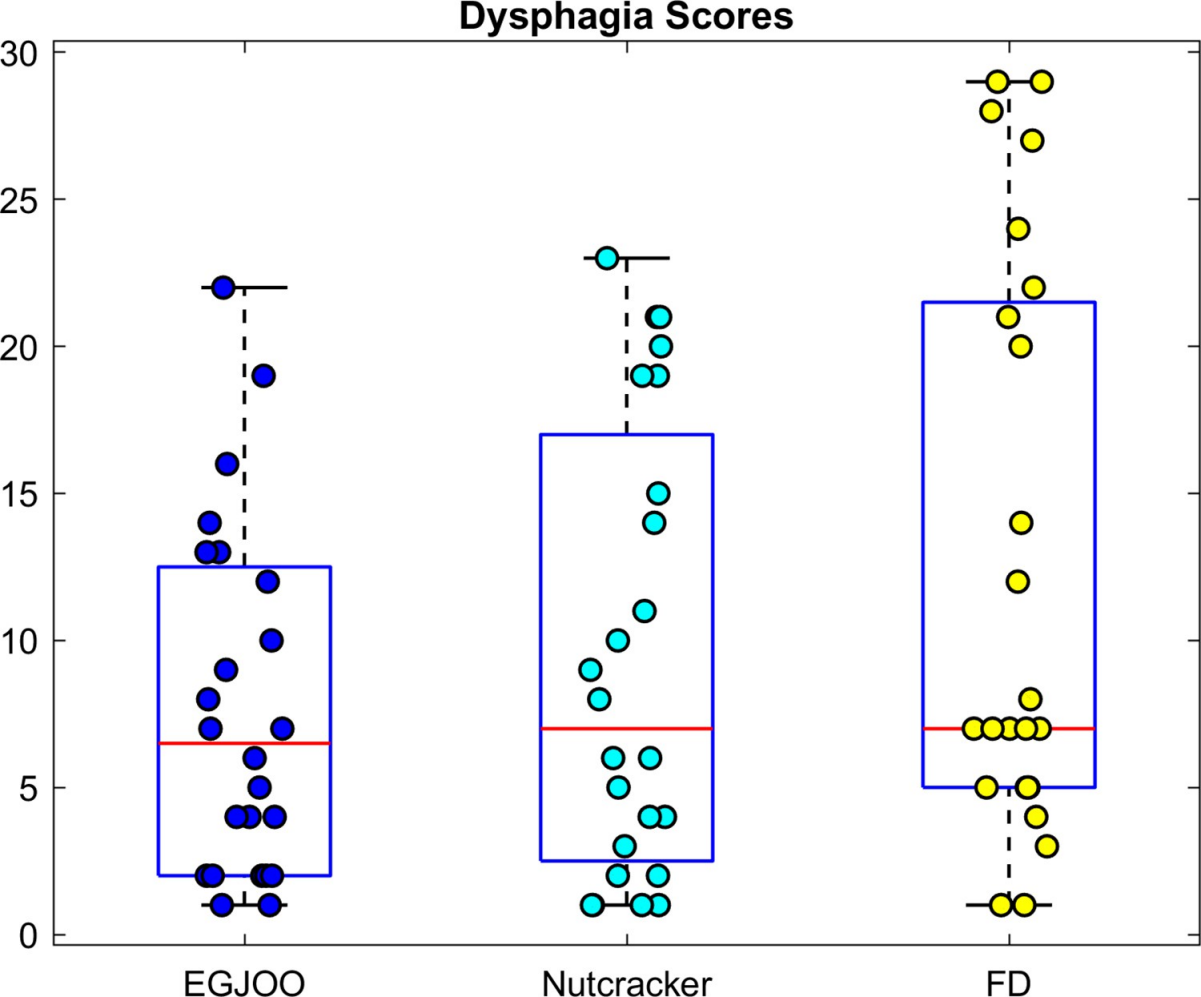
Dysphagia scores in 3 group of patients are not different. EGJOO = esophagogastric junction outflow obstruction, FD = functional dysphagia, and NC = nutcracker esophagus.

There were no significant differences between the 3 patient groups with regards to dysphagia scores (Fig 6). These values were 7.7±6 for EGJOO, 12.4±9.7 for FD and 9.4±7.6 for Nutcracker group (EGJOO vs FD, P = 0.118; EGJOO vs Nutcracker, P = 1; FD vs Nutcracker, P = 0.487). There were 2 patients in NC esophagus group with no dysphagia, their presenting symptom was non-cardiac chest pain. The distension profile of these 2 patients was different; their amplitude and duration of distension, and the temporal relation between distension and contraction resembled normal subjects. A further analysis to study whether there exists any (linear) correlation between patient’s dysphagia score and objective parameters was carried out. Except for the EGJOO group, which showed a weak correlation (*r* = 0.32, Kendall correlation) in 4^th^ segment with peak distension amplitude, no other significant correlation was found.

## Discussion

Described by Bayless and Starling, 120 years ago [21, 22], initial inhibition/relaxation followed by excitation/contraction are the hallmarks of peristalsis, “law of intestine” throughout the gastrointestinal tract, including esophagus. Electrophysiology studies reveal hyperpolarization followed by depolarization of the muscle cell membrane, which are correlates of relaxation and contraction phase of peristalsis, respectively [23, 24]. Discovered by L.D. Harris [25], and improvements by many investigators [26, 27] the technique of infusion manometry allowed accurate measurements of the contraction phase of esophageal peristalsis in animals and humans. The HRM technique that utilizes closely spaced, high fidelity pressure sensors are an almost perfect technique to record the contraction phase of peristalsis. Swallow-induced relaxation of LES, which begins soon after the onset of a swallow is related to the activation of inhibitory motor neurons. Unlike LES, manometry does not record any baseline tone in the esophagus and hence it cannot record the inhibition phase of esophageal peristalsis. However, swallow-induced inhibition can be recorded by manometry technique, using multiple swallows at short intervals (< 2 second apart), where each subsequent swallow inhibits the preceding esophageal contraction [28]. Sifrim used an ingenious method of artificial high-pressure zones in the esophagus to demonstrate normal and abnormal inhibitory phase of peristalsis in healthy subjects and patients with spastic esophageal motor disorders, respectively [29, 30]. The purpose of initial inhibition of peristalsis is to allow luminal distension during peristalsis.

Our earlier studies validated impedance methodology to measure the luminal CSA. In normal subjects, important characteristics of esophageal distension waveforms, as bolus travels through the esophagus during swallow-induced peristalsis are, 1) the amplitude and duration of distension increases from proximal to distal esophagus, 2) bolus travels in the shape of an “American Football” along the entire length of esophagus, 3) peak distension travels sequentially along the esophagus, and 4) there is a close temporal correlation between peak distension and onset/peak of contraction [6, 10]. We found important differences between patients and normal. In patients, 1) bolus arrives in the distal esophagus faster than in normal subjects, 2) amplitude of distension is smaller and, 3) distension waveform is multi-peaked, which translates into fragmented bolus movement in the esophagus. Following a swallow, the pharyngeal pump pushes bolus into distal esophagus quickly, much ahead of the contraction wave. As per Poiseuille law of fluid flow, bolus velocity in a tube is inversely related to the luminal CSA, and hence bolus will move much faster in a narrow than a wide lumen esophagus, resulting in a smaller T1, which is what we found. Smaller amplitude of distension also implies a narrow esophagus in the patients. On barium swallow studies, fragmentation of bolus during its transit through the esophagus (corkscrew or rosary-bead esophagus) is a hallmark of the spastic motor disorders. Our findings imply that the impedance method of recording luminal CSA and bolus transit is a sensitive marker of the abnormalities of fluid flow during the inhibitory phase of peristalsis.

Based on the HRM recordings and Chicago classification [7] of esophageal motility disorders, which is based on the parameters of LES relaxation and esophageal contractions, we studied 3 distinct group of patients with dysphagia, i.e., NC esophagus, EGJOO, and FD. All of the above subjects have sequential contraction (peristalsis) in the esophagus. Our findings of esophageal distension abnormalities in patients with functional dysphagia and nutcracker esophagus have been reported earlier. The novel aspect of our current study is our findings in patients with EGJOO and comparison between the various groups. We found abnormalities of luminal distension and bolus flow in all the 3 patient groups with dysphagia symptom that were similar, both qualitatively and quantitatively. We suspect that the fundamental abnormality in these three patient groups with dysphagia manifests in the distension phase of peristalsis, and not necessarily in the contraction phase, except in the nutcracker esophagus who have supernormal contraction phase of peristalsis. With regards to the mechanism of reduced distension there are several possibilities, i.e., 1) impaired inhibitory innervation of the esophagus, similar to what is seen in patients with achalasia esophagus and diffuse esophageal spasm, 2) low compliance of the esophageal wall, which may be related to the muscle hypertrophy seen in patients with nutcracker esophagus [31], and 3) discoordination between circular and longitudinal muscle contraction during peristalsis [32]. Our study does not reveal which of the above is the cause of distension abnormality in our patient groups.

The dysphagia score was similar among 3 patient groups. Lack of correlation between the dysphagia score and any of the distension/contraction parameters that we measured requires an explanation. Dysphagia was an important symptom in all of patient groups, except for 2 patients in the NC esophagus. Interestingly, these two NC patients revealed normal distension phase of peristalsis. Lack of correlation between esophageal symptoms and esophageal contraction abnormalities has been described before [33]. Furthermore, a recent study found that dysphagia score in patients with achalasia esophagus and EGJOO are not different, which is surprising given significantly greater manometric abnormalities in achalasia esophagus (impaired LES relaxation and loss of peristalsis) than EGJOO (only impaired LES relaxation [34]. We propose/suspect that the normal contraction phase of peristalsis (seen in EGJOO and FD) or supernormal contraction phase (seen in nutcracker esophagus) results in a scenario where esophageal contraction is pushing the bolus through a narrow tube and it is the latter that results in dysphagia sensation. Above is not dissimilar to air moving through a narrow trachea-bronchial tube (bronchospasm) and causing dyspnea sensation.

There are few limitations of our study. First, normal subjects were younger than the patient groups in our study. Second, we may not have used all HRM criteria suggested by Chicago Classification 4.0 to divide patients into various HRM groups, e.g., our definition of nutcracker esophagus and EGJOO are slightly different than suggested. Third, we did not have information in regards to muscle relaxant medications, as that was not the criteria for inclusion in the study. Our focus was the assessment of dysphagia. Furthermore, Chicago Classification criteria are based on consensus rather than any pathophysiology or patient outcomes. Finally, we did not perform endoflip testing to assess distensibility of the esophago-gastric junction (EGJ) in our patients [35]. Withstanding above limitations, our findings suggest that impaired distension of the esophagus during peristalsis is an important finding in patients with dysphagia, with subtle or no contraction abnormalities of the esophagus. Whether addressing abnormalities of the distension phase of peristalsis, using pharmacologic approaches, esophageal dilation, and possibly some sort of surgery will impact dysphagia symptom requires further studies.

## Supporting information

S1 Fig HRMZ acquisition protocol. (TIF)

S1 Video Video of the temporal distension contraction patterns of Fig 4 during a 10cc, 0.5N saline swallow in four groups. (MP4)
