# Supplementary figures and images for: Rhythmic contraction but arrhythmic distension of esophageal peristaltic reflex in patients with dysphagia
Source: PLoS One. 2022 Jan 24;17(1):e0262948. doi: 10.1371/journal.pone.0262948 (PMC8786162; doi:10.1371/journal.pone.0262948)

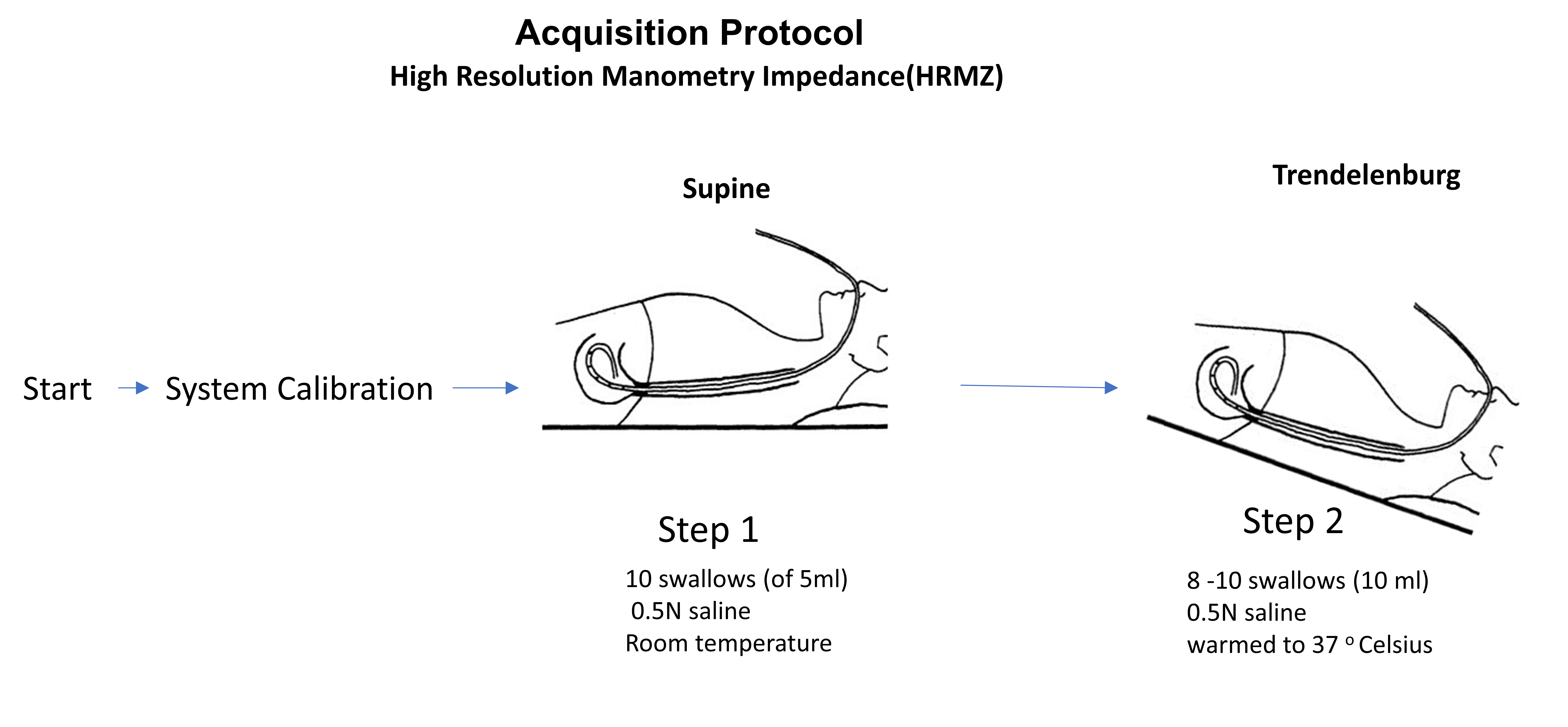

Supplement: S1 Fig — (TIF) [file pone.0262948.s001.tif]
